# 2010年-2015年SEER数据库肺腺鳞癌患者临床特征及预后分析

**DOI:** 10.3779/j.issn.1009-3419.2018.08.14

**Published:** 2018-08-20

**Authors:** 成 詹, 天 江, 晓冬 杨, 卫刚 郭, 黎杰 谭

**Affiliations:** 200032 上海，复旦大学附属中山医院胸外科 Department of Thoracic Surgery, Zhongshan Hospital, Fudan University, Shanghai 200032, China

**Keywords:** 肺肿瘤, 腺鳞癌, 列线图, SEER数据库, Lung neoplasms, Adenosquamous carcinoma, Nomogram, SEER database

## Abstract

**背景与目的:**

肺癌发病率和死亡率均位居所有恶性肿瘤的第一，严重影响人类健康。非小细胞肺癌（non-small cell lung cancer, NSCLC）中常见病理类型为腺癌和鳞癌，临床研究和关注较多，而肺腺鳞癌是一种较为罕见的肺癌病理类型，其临床特征及预后相关因素尚未完全明确。本研究即对肺腺鳞癌的临床特征及预后进行分析，并构建了列线图来预测患者的预后。

**方法:**

我们纳入了2010年-2015年美国SEER（Surveillance, Epidemiology, and End Results）数据库中的肺腺鳞癌数据，与同期的肺腺癌和肺鳞癌的临床特征和预后进行了比较。随后我们采用单因素和多因素分析研究了肺腺鳞癌患者预后的独立相关因素，以此构建了列线图并进行了验证。

**结果:**

我们一共入组了肺腺鳞癌患者1, 453例。与同期的肺腺癌和肺鳞癌患者相比较，肺腺鳞癌患者在大多数变量中的分布情况均介于肺腺癌和鳞癌之间，其预后也优于肺鳞癌但差于肺腺癌患者。多因素分析发现，年龄、分化程度、肿瘤-淋巴结-转移（tumor-node-metastasis, TNM）、手术和化疗是患者预后的独立影响因素（*P*均 < 0.001）。我们以此构建了列线图，其C-index为0.783（0.767-0.799），区分度检验和一致性检验均表明这一列线图可以有效地预测患者预后。

**结论:**

肺腺鳞癌具有独特的临床病理和预后特征。年龄、分化、T、N、M、手术和化疗状况是肺腺鳞癌患者预后的独立预测因素。我们以此构建的列线图可以较好地预测患者预后。

## Abstract (en)

**Background and objective:**

The incidence and the mortality of lung cancer rank first among all malignant tumors and it seriously affects human health. The common types of non-small cell lung cancer (NSCLC) are adenocarcinoma and squamous carcinoma with clinical research and more attention, while adenosquamous carcinoma is a rare pathological subtype of lung cancer, which clinical features and prognostic factors are not yet fully understood. The purpose of this study is to analyze the clinical features and prognosis of lung adenosquamous carcinoma, and construct a nomogram to predict the patients' prognosis.

**Methods:**

We obtained the data of adenosquamous carcinoma patients diagnosed between 2010 and 2015 in the Surveillance, Epidemiology, and End Results (SEER) database of the United States, and compared their clinical features and prognosis with those of lung adenocarcinoma and lung squamous cell carcinoma patients in the same period. Then we used univariate and multivariate analyses to explore the independent prognostic factors of adenosquamous carcinoma. Finally, we constructed and validated a nomogram to visually predict the outcomes of lung adenosquamous carcinoma.

**Results:**

1, 453 patients with lung adenosquamous carcinoma were finally included. Compared with patients with lung adenocarcinoma and lung squamous cell carcinoma, the distributions of lung adenocarcinoma patients in most of the variables were medium between lung adenocarcinoma and squamous cell carcinoma. The prognosis of adenosquamous carcinoma was better than that of lung squamous cell carcinoma, but worse than that of lung adenocarcinoma. Multivariate analysis showed that age, differentiation, tumor-node-metastasis (TNM), surgery, and chemotherapy were independent prognostic factors (all *P* were less than 0.001). We constructed a nomogram with a C-index of 0.783 (0.767-0.799). The distinction test and consistency test showed that the nomogram could predict the patient's prognosis effectively.

**Conclusion:**

Lung adenosquamous carcinoma has unique clinical, pathological, and prognostic characteristics. Age, differentiation, T, N, M, surgery, and chemotherapy status are independent predictors of prognosis in patients with adenosquamous carcinoma. Our nomogram can efficiently predict the prognosis of patients.

肺癌发病率和死亡率均位居所有恶性肿瘤的第一位，2012年世界发病人数约182万人，死亡人数约159万人，严重地影响了全人类的健康^[1, 2]^。腺癌和鳞癌是肺癌的最主要的病理类型，得到了广泛的研究与关注。但在腺癌和鳞癌之外，还存在着许多并不常见的肺癌病理类型，它们的临床特征和预后因素仍不明确。

腺鳞癌是其中一类较为罕见的肺癌病理类型，据报道其仅占所有肺癌的0.4%-4%^[3, 4]^。根据当前的分类方法，肿瘤必须含有至少10%的腺癌或鳞癌成分时才能诊断为腺鳞癌^[5-7]^。肺腺鳞癌有着其独特的临床病理和预后特征。在本研究中，我们利用美国SEER（Surveillance, Epidemiology, and End Results）数据库的肺腺鳞癌病例数据，分析了肺腺鳞癌与肺腺癌、肺鳞癌在临床特征上的差别，并研究了肺腺鳞癌预后的相关因素。我们进一步基于生存分析的结果制作了肺腺鳞癌患者的列线图，以更好地预测患者的预后。

## 1 材料与方法

### 1.1 数据来源

我们通过SEER*Stat软件（v8.3.5，https://seer.cancer.gov/seerstat/）从SEER数据库（http://seer.cancer.gov/）中下载了2010年-2015年的所有原发性肺癌患者数据。排除标准为：①有其他部位肿瘤史；②分化、分期、治疗方式等未知；③病理类型非腺鳞癌、腺癌或者鳞癌（图 1）。

**1:**
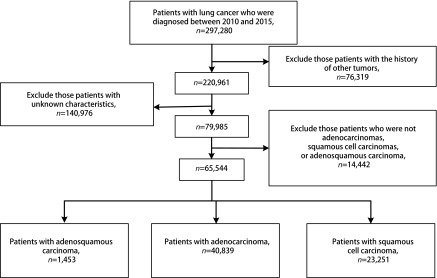
患者筛选流程图 Flow diagram of selecting process

我们提取并分析了患者的种族、年龄、性别、肿瘤位置、分化程度、肿瘤-淋巴结-转移（tumor-node-metastasis, TNM）、分期、手术、放疗、化疗、保险、婚姻状况以及生存状态和时间等变量，并且我们按照第八版美国癌症联合会（American Joint Committee on Cancer, AJCC）肺癌分期方案重新整理了所有患者的T、N、M以及分期。

### 1.2 统计分析

在肺腺鳞癌、腺癌和鳞癌之间的临床特征比较中，对于种族、肿瘤位置等多分类变量的比较采用卡方检验、对于性别、分化、T、N、M、分期、手术、放疗、化疗、保险、婚姻状况等二分类变量或者有序变量的比较采用秩和检验，对于年龄这一定量变量的比较采用方差分析。在肺腺鳞癌的预后因素分析，我们采用*Kaplan-Meier*分析和*Log-rank*检验进行单因素分析，采用*Cox*模型进行多因素分析。以上分析均采用SPSS（v25）进行，均为双侧检验，*P* < 0.05为差异具有统计学意义。

我们采用R语言（v4.3.4）进行列线图的制作和检验，用到的主要软件包为rms和Hmisc^[8, 9]^。

## 2 结果

### 2.1 肺腺鳞癌、腺癌和鳞癌之间的临床特征比较

如图 1所示，经筛选后，我们一共入组同期的肺腺鳞癌患者1, 453例，腺癌患者40, 839例和鳞癌患者23, 251例。肺腺鳞癌患者数量仅为腺癌患者数量的3.6%和鳞癌患者数量的6.3%。经统计分析发现，肺腺鳞癌、腺癌和鳞癌患者的种族、性别、年龄、肿瘤位置、分化程度、T、N、M以及总分期、手术和放疗、以及婚姻和保险状况均存在显著差异（表 1）。腺鳞癌的种族、性别、年龄、肿瘤位置、分期以及放疗等变量分布特征介于腺癌和鳞癌中间，但腺鳞癌的分化程度要低于腺癌和鳞癌，接受手术的比例要高于腺癌和鳞癌。腺鳞癌男性患者比例略多于女性患者。腺鳞癌好发位置为右上肺叶和左上肺叶，占比分别为34.1%和28.7%。低分化患者占所有腺鳞癌患者的65.2%。生存分析显示：腺鳞癌患者的预后也介于腺癌患者和鳞癌患者之间，中位生存时间分别为22.0（19.3-24.7）、32.0（31.1-32.9）、17.0（16.5-17.5）个月，差异存在统计学意义（*P* < 0.001）（图 2）。

**1:** 肺腺鳞癌、肺腺癌和肺鳞癌患者临床病理特征比较 Comparison of the clinipathological characteristics of lung adenosquamous carcinoma with those of adenocarcinoma and squamous cell carcinoma

Characteristics	Adenosquamous carcinoma	Adenocarcinoma	Squamous cell carcinoma	*P*
*n*	1, 453	40, 839	23, 251	
Race				< 0.001
White	1, 186 (81.6%)	32, 309 (79.1%)	19, 501 (83.9%)	
Black	141 (9.7%)	4, 623 (11.3%)	2, 542 (10.9%)	
Others	126 (8.7%)	3, 907(9.6%)	1, 208 (5.2%)	
Gender				< 0.001
Male	781 (53.8%)	18, 760 (45.9%)	14, 443 (62.1%)	
Female	672 (46.2%)	22, 061 (54.1%)	8, 802 (37.9%)	
Age (Mean±SD, yr)	69.2±10.1	67.0±11.0	69.9±9.6	< 0.001
Marriage				< 0.001
No	614 (42.3%)	18, 065 (44.2%)	11, 091 (47.7%)	
Yes	839 (57.7%)	22, 774 (55.8%)	12, 160 (52.3%)	
Insurance				< 0.001
No	24 (1.7%)	1, 091 (2.7%)	506 (2.2%)	
Yes	1, 429 (98.3%)	39, 748 (97.3%)	22, 745 (97.8%)	
Site				< 0.001
Left upper lobe	417 (28.7%)	10, 527 (25.8%)	6, 784 (29.2%)	
Left lower lobe	205 (14.1%)	5, 669 (13.9%)	3, 286 (14.1%)	
Right upper lobe	495 (34.1%)	14, 616 (35.8%)	7, 540 (32.4%)	
Right middle lobe	52 (3.6%)	2, 437 (6.0%)	950 (4.1%)	
Right lower lobe	261 (18.0%)	7, 058 (17.3%)	4, 322 (18.6%)	
Overlapping lobes	23 (1.6%)	532 (1.3%)	369 (1.6%)	
Grade				< 0.001
Well differentiated	17 (1.2%)	7, 591 (18.6%)	709 (3.0%)	
Moderately differentiated	461 (31.7%)	15, 130 (37.0%)	9, 941 (42.8%)	
Poorly differentiated	947 (65.2%)	17, 563 (43%)	12, 396 (53.3%)	
Undifferentiated	28 (1.9%)	555 (1.4%)	205 (0.9%)	
T stage				< 0.001
T1	373 (25.7%)	13, 569 (33.2%)	4, 957 (21.3%)	
T2	593 (40.8%)	13, 408 (32.8%)	8, 454 (36.4%)	
T3	281 (19.3%)	7, 291 (17.9%)	5, 394 (23.2%)	
T4	206 (14.2%)	6, 571 (16.1%)	4, 446 (19.1%)	
N stage				< 0.001
N0	753 (51.8%)	22, 411 (54.9%)	11, 915 (51.2%)	
N1	190 (13.1%)	3, 979 (9.7%)	2, 590 (11.1%)	
N2	402 (27.7%)	10, 866 (26.6%)	6, 914 (29.7%)	
N3	108 (7.4%)	3, 583 (8.8%)	1, 832 (7.9%)	
M stage				< 0.001
M0	1, 069 (73.6%)	27, 360 (67.0%)	17, 249 (74.2%)	
M1	384 (26.4%)	13, 479 (33.0%)	6, 002 (25.8%)	
Stage				< 0.001
Ⅰ	552 (38.0%)	16, 246 (39.8%)	7, 971 (34.3%)	
Ⅱ	230 (15.8%)	4, 565 (11.2%)	3, 413 (14.7%)	
Ⅲ	287 (19.8%)	6, 549 (16.0%)	5, 865 (25.2%)	
Ⅳ	384 (26.4%)	13, 479 (33.0%)	6, 002 (25.8%)	
Surgery				< 0.001
No	581 (40.0%)	19, 776 (48.4%)	14, 153 (60.9%)	
Yes	872 (60.0%)	21, 063 (51.6%)	9, 098 (39.1%)	
Radiotherapy				< 0.001
No	971 (66.8%)	27, 519 (67.4%)	13, 077 (56.2%)	
Yes	482 (33.2%)	13, 320 (32.6%)	10, 174 (43.8%)	
Chemotherapy				0.112
No/Unknown	854 (58.8%)	24, 441 (59.8%)	13, 727 (59.0%)	
Yes	599 (41.2%)	16, 398 (40.2%)	9, 524 (41.0%)	

**2:**
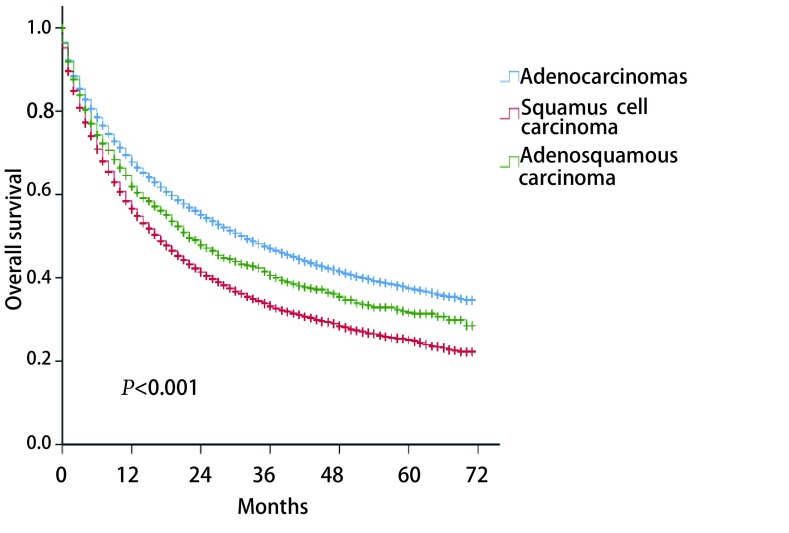
肺腺鳞癌、肺腺癌和肺鳞癌患者生存比较 The survival curves of lung adenosquamous carcinoma, adenocarcinoma, and squamous cell carcinoma

### 2.2 腺鳞癌的预后因素分析

我们采用单因素分析对肺鳞腺癌患者的可能的预后因素进行研究发现，年龄、分化程度、T、N、M、总分期、手术、放疗和化疗与患者预后的相关性存在统计学意义（*P*均 < 0.001）（图 3、图 4、图 5）。年龄越大、分化程度越低、T、M、M及总分期越高，患者的预后越差。另外，单因素分析结果显示，接受手术的患者预后较未接受手术的患者预后更好，而接受放疗和化疗的患者分别较未接受的患者预后更差。种族（*P*=0.127）、性别（*P*=0.216）、婚姻（*P*=0.065）、保险状况（*P*=0.205）和肿瘤位置（*P*=0.110）与预后的相关性无显著统计学意义。

**3:**
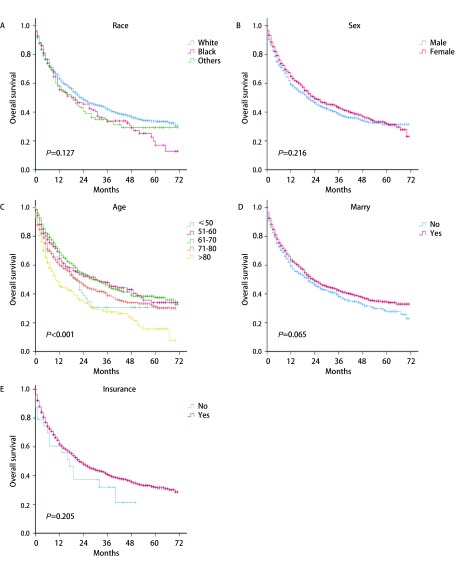
种族、性别、年龄、婚姻和保险状况与肺腺鳞癌患者预后分析。A：种族；B：性别；C：年龄；D：婚姻状况；E：保险状况 Survival analyses of patients with lung adenosquamous carcinoma according to race (A), gender (B), age (C), marriage (D), and insurance status (E)

**4:**
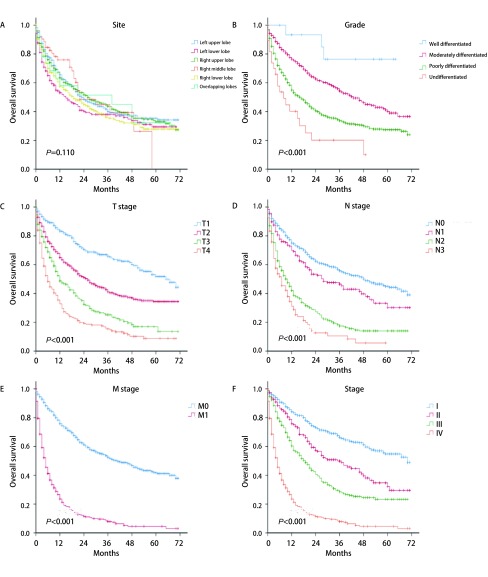
肿瘤位置、分化、T、N、M、总分期与肺腺鳞癌患者预后分析。A：肿瘤位置；B：分化；C：T分期；D：N分期；E：M分期；F：总分期 Survival analyses of patients with lung adenosquamous carcinoma according to site (A), grade (B), T stage (C), N stage (D), M stage (E), and TNM stage (F). TNM: tumor-node-matastasis

**5:**
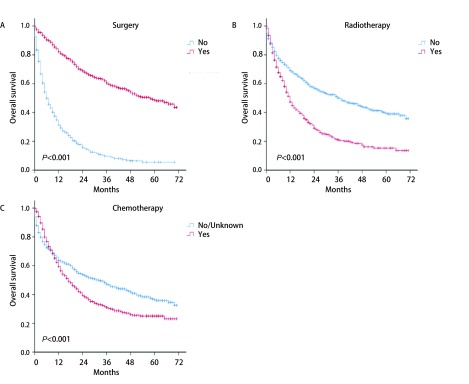
手术、放疗和化疗状况与肺腺鳞癌患者预后分析。A：手术；B：放疗；C：化疗 Survival analyses of patients with lung adenosquamous carcinoma according to surgery (A), radiotherapy (B), and chemotherapy (C)

进一步将这些在单因素分析中具有统计学意义的变量纳入多变量分析后发现，年龄、分化程度、T、N、M、手术和化疗是患者预后的独立影响因素（*P*均 < 0.001）（表 2）。多因素分析结果同样显示，患者的年龄越大、分化程度越低、T、M及总分期越高，预后越差。但N1、N2、N3对患者预后的影响接近，与N0相比较其OR分别为1.474（1.110-1.958）、1.512（1.237-1.849）、1.519（1.192-1.937）。接受手术的患者预后显著优于未手术的患者，其OR为0.335（0.273-0.410）。与单因素分析结果不同，多因素分析结果显示接受化疗是较好的预后因素，OR为0.607（0.513-0.718），意味着排除其他因素的影响后，接受化疗的患者相对未接受化疗的患者有着更好的预后。同时结果显示放疗并不是一个独立的预后影响因素（*P*=0.500）。因为总分期与T、N、M分期有关，不是一个独立的变量，因此未纳入到多因素分析中。

**2:** 多因素分析肺腺鳞癌患者预后的独立影响因素 Cox proportional hazards regression analysis for patients with lung adenosquamous carcinoma

Characteristics	OR (95%CI)	*P*
Age (yr)		< 0.001
≤50	Reference	
51-60	0.998 (0.693-1.439)	0.993
61-70	1.131 (0.806-1.587)	0.476
71-80	1.547 (1.101-2.172)	0.012
> 80	1.779 (1.227-2.579)	0.002
Grade		< 0.001
Well differentiated	Reference	
Moderately differentiated	4.392 (1.398-13.801)	0.011
Poorly differentiated	5.517 (1.764-17.26)	0.003
Undifferentiated	12.945 (3.808-44.006)	< 0.001
T stage		< 0.001
T1	Reference	
T2	1.572 (1.265-1.953)	< 0.001
T3	1.743 (1.359-2.235)	< 0.001
T4	1.806 (1.387-2.353)	< 0.001
N stage		< 0.001
N0	Reference	
N1	1.474 (1.110-1.958)	< 0.001
N2	1.512 (1.237-1.849)	< 0.001
N3	1.519 (1.192-1.937)	0.007
M stage		< 0.001
M0	Reference	
M1	2.284 (1.898-2.749)	< 0.001
Surgery		< 0.001
No	Reference	
Yes	0.335 (0.273-0.410)	< 0.001
Radiotherapy		0.500
No	Reference	
Yes	0.943 (0.796-1.118)	0.500
Chemotherapy		< 0.001
No/Unknown	Reference	
Yes	0.607 (0.513-0.718)	< 0.001

### 2.3 列线图的制作及检验

我们基于以上的患者预后的独立预测因素成功构建了列线图（图 6A），依据患者的年龄、分化、T、N、M、手术和化疗状况，我们可以可视化地计算出患者的3年和5年生存率。经区分度检验，这一列线图的C-index为0.783（0.767-0.799）。一致性检验显示，列线图所预测的3年、5年生存率与实际的3年、5年生存率有着很好的一致性，一致性曲线斜率接近于1（图 6B，图 6C）。

**6:**
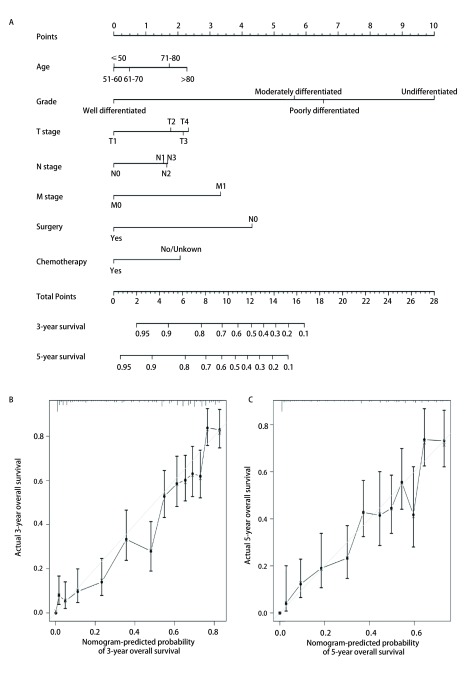
肺腺鳞癌患者的列线图以及一致性检验。A：列线图；B：3年生存率的一致性检验；C：5年生存率的一致性检验 The nomogram of lung adenosquamous carcinoma and the calibration curves. A: the nomogram; B: calibration curve predicting 3-year survival; C: calibration curve predicting 5-year survival

## 3 讨论

在本研究中，我们对美国SEER数据库中的肺腺鳞癌患者数据进行了深入的分析，发现肺腺鳞癌患者在种族、年龄、性别等多个方面均介于肺腺癌和鳞癌之间，其预后也优于肺鳞癌但比肺腺癌患者差。我们发现年龄、分化程度、T、N、M、手术和化疗是肺腺鳞癌患者预后的独立影响因素并以此绘制了列线图。区分度检验和一致性检验表明这一列线图能有效地预测患者3年和5年生存概率。

在我们的研究中，我们发现肺腺鳞癌的预后介于肺腺癌和肺鳞癌患者之间。但在大多数研究中，肺腺鳞癌的预后均差于肺腺癌和肺鳞癌^[4, 10-12]^。这些研究中往往比较的是手术后的肺腺鳞癌、腺癌和鳞癌患者，这会给研究结果带来一定的偏倚。Wang等^[13]^对肺腺鳞癌、腺癌和鳞癌患者的肿瘤特异性生存进行初步比较时发现肺腺鳞癌患者预后要优于肺腺癌和鳞癌患者。但Wang等^[13]^进一步分别采用倾向性匹配消除肺腺鳞癌和肺腺癌、以及肺腺鳞癌和肺鳞癌之间临床特征的差异后再进行比较，发现同样条件下的肺腺鳞癌患者预后较肺腺癌和肺鳞癌患者差。

手术和化疗是肺腺鳞癌患者的主要治疗方式，在我们的研究中手术和化疗也是患者预后独立的影响因素，接受手术和化疗的患者预后会更好，不管是单因素分析还是多因素分析，手术的患者预后更佳，这和患者的分期相关，一般来说，能手术的患者分期偏早。而化疗在单因素分析中提示接受化疗的患者预后较未接受化疗的患者差，多因素分析排除其他因素后提示化疗对于非腺鳞癌仍旧是较好的预后因素。靶向治疗是肺腺鳞癌未来的发展方向，但目前对肺腺鳞癌患者的靶向治疗方案及效果鲜有报道^[14]^。肺腺鳞癌的基因突变也具有不同于肺腺癌和肺鳞癌的特征。Powrozek等^[15]^和Shiozawa等^[16]^分别报道28.6%和23.7%的肺腺鳞癌患者中存在*EGFR*突变。Vassella等^[17]^报道30%和25%的肺腺鳞癌患者分别存在*EGFR*和*PI3K*信号通路的突变。Liu等^[18]^报道48.1%的肺腺鳞癌患者肿瘤组织中PD-L1表达阳性。

SEER数据库是美国基于人群的肿瘤流行病学数据库，覆盖了美国28%左右的人群，包含了自1973年以来的几十万例肺癌病例以及详细的临床和预后信息，对肺癌及其他肿瘤的研究有着极大的帮助^[19, 20]^。通过对SEER数据库中整个人群中的病例进行分析，可以有效地避免患者来自单一机构所给研究带来的偏倚。但SEER数据库中缺乏影像学、吸烟史、基因突变、肿瘤标志物、以及详细的治疗方式尤其是化疗方案等信息，我们的研究中也未涉及到这些因素对肺腺鳞癌患者预后的影响。而这些因素可能显著地作用于患者的预后。

肺腺鳞癌具有与肺腺癌、鳞癌不同的临床病理和预后特征。年龄、分化、T、N、M、手术和化疗状况是肺腺鳞癌患者预后的独立预测因素。我们以此构建的列线图可以较好地预测患者的3年和5年生存率。

## References

[1] Torre LA, Bray F, Siegel RL (2015). Global cancer statistics, 2012. CA Cancer J Clin.

[2] Allemani C, Matsuda T, Di Carlo V (2018). Global surveillance of trends in cancer survival 2000-14 (CONCORD-3): analysis of individual records for 37 513 025 patients diagnosed with one of 18 cancers from 322 population-based registries in 71 countries. Lancet.

[3] Shimoji M, Nakajima T, Yamatani C (2011). A clinicopathological and immunohistological re-evaluation of adenosquamous carcinoma of the lung. Pathol Int.

[4] Zhu L, Jiang L, Yang J (2018). Clinical characteristics and prognosis of patients with lung adenosquamous carcinoma after surgical resection: results from two institutes. J Thorac Dis.

[5] Zhao WJ, Wang X, Ma KW (2017). Progression of diagnosis and treatment of adenosquamous lung cancer. Zhonghua Zhong Liu Za Zhi.

[6] Travis WD, Brambilla E, Noguchi M (2011). International association for the study of lung cancer/american thoracic society/european respiratory society international multidisciplinary classification of lung adenocarcinoma. J Thorac Oncol.

[7] Travis WD, Brambilla E, Burke AP (2015). Introduction to The 2015 World Health Organization Classification of Tumors of the Lung, Pleura, Thymus, and Heart. J Thorac Oncol.

[8] Wang S, Ma K, Chen Z (2018). A nomogram to predict prognosis in malignant pleural mesothelioma. World J Surg.

[9] Sun F, Ma K, Yang X (2017). A nomogram to predict prognosis after surgery in early stage non-small cell lung cancer in elderly patients. Int J Surg.

[10] Maeda H, Matsumura A, Kawabata T (2012). Adenosquamous carcinoma of the lung: surgical results as compared with squamous cell and adenocarcinoma cases. Eur J Cardiothorac Surg.

[11] Filosso PL, Ruffini E, Asioli S (2011). Adenosquamous lung carcinomas: a histologic subtype with poor prognosis. Lung Cancer.

[12] Gawrychowski J, Brulinski K, Malinowski E (2005). Prognosis and survival after radical resection of primary adenosquamous lung carcinoma. Eur J Cardiothorac Surg.

[13] Wang J, Lian B, Ye L (2018). Clinicopathological characteristics and survival outcomes in adenosquamous carcinoma of the lung: a population-based study from the SEER database. Oncotarget.

[14] Jiang NY, Pan HM, Li D (2017). Research progression of lung adenosquamous carcinoma. Ai Zheng Jin Zhan.

[15] Powrozek T, Krawczyk P, Ramlau R (2014). *EGFR* gene mutations in patients with adenosquamous lung carcinoma. Asia Pac J Clin Oncol.

[16] Shiozawa T, Ishii G, Goto K (2013). Clinicopathological characteristics of *EGFR* mutated adenosquamous carcinoma of the lung. Pathol Int.

[17] Vassella E, Langsch S, Dettmer MS (2015). Molecular profiling of lung adenosquamous carcinoma: hybrid or genuine type?. Oncotarget.

[18] Liu Y, Dong Z, Jiang T (2018). Heterogeneity of PD-L1 expression among the different histological components and metastatic lymph nodes in patients with resected lung adenosquamous carcinoma. Clin Lung Cancer.

[19] Yang X, Zhan C, Li M (2018). Lobectomy versus sublobectomy in metachronous second primary lung cancer: a propensity-score study. Ann Thorac Surg.

[20] Yang X, Sun F, Chen L (2017). Prognostic value of visceral pleural invasion in non-small cell lung cancer: A propensity score matching study based on the SEER registry. J Surg Oncol.

